# Vaginal Microbiota and the Use of Probiotics

**DOI:** 10.1155/2008/256490

**Published:** 2009-03-29

**Authors:** Sarah Cribby, Michelle Taylor, Gregor Reid

**Affiliations:** ^1^Canadian Research and Development Centre for Probiotics, Lawson Health Research Institute, 268 Grosvenor Street, London, ON, Canada N6A4V2; ^2^Department of Microbiology and Immunology, University of Western Ontario, London, ON, Canada N6A4V2

## Abstract

The human vagina is inhabited by a range of microbes from a pool of over 50 species. Lactobacilli are the most common, particularly in healthy women. The microbiota can change composition rapidly, for reasons that are not fully clear. This can lead to infection or to a state in which organisms with pathogenic potential coexist with other commensals. The most common urogenital infection in premenopausal women is bacterial vaginosis (BV), a condition characterized by a depletion of lactobacilli population and the presence of Gram-negative anaerobes, or in some cases Gram-positive cocci, and aerobic pathogens. Treatment of BV traditionally involves the antibiotics metronidazole or clindamycin, however, the recurrence rate remains high, and this treatment is not designed to restore the lactobacilli. In vitro studies have shown that *Lactobacillus* strains can disrupt BV and yeast biofilms and inhibit the growth of urogenital pathogens. The use of probiotics to populate the vagina and prevent or treat infection has been considered for some time, but only quite recently have data emerged to show efficacy, including supplementation of antimicrobial treatment to improve cure rates and prevent recurrences.

## 1. THE MICROBIOTA OF THE VAGINA

The microbial species that inhabit the vaginal tract play an important role in the maintenance of health, and prevention of infection. Over 50 microbial species have been recovered from the vaginal tract [1–3]. These species do not exist independently, and studies in vitro and in humans have shown that a multispecies microbiota, usually associated with bacterial vaginosis (BV), are present in dense biofilms [4–7], while a lactobacilli dominant microbiota can be sparsely distributed on the epithelium [4, 5, 8]. In comparison, the gut is populated with more than 800 species of microbes, the majority of which are excreted in feces, and a number of which are well equipped to be pathogenic. Despite the close proximity of the vagina to the anus, the diversity of microbes present in the vagina is much lower than in the gut. The reason for this lower diversity is still unclear, but may involve poor receptivity of the vagina, different nutrient availability compared to the gut, and competition with indigenous organisms. Some species found in the gut, such as *E. coli* and *Streptococcus,* can also be found in the vagina, indicating the proper receptors, nutrients, and oxygen tension are present for these organisms to grow.

Different methodologies are being used to identify the composition of the vaginal microbiota. Each has its strengths and weaknesses. Culture-based methods allow strains to be identified and used for further experimentation. However, as there remains a major defect in our ability to grow many bacterial species, we must rely on nonculture methods to identify the breadth of vaginal microbiota. This has been achieved by analyzing their ribosomal DNA sequences [3, 9], using a combination of PCR and denaturing gel gradient electrophoresis (DGGE) [2, 5, 10–12], and by using degenerate, universal polymerase chain reaction primers to amplify an approximately 555 base-pair regions of the universal chaperonin-60 gene [13].

The species that are present in the vaginal mucosa vary between premenopausal woman and those who have gone through menopause. The microbiota of healthy premenopausal woman is generally dominated by *Lactobacillus* species, the most common of which are *L. iners*, *L. crispatus*, *L. gasseri*, *L. jenesenii,* followed by *L. acidophilus, L. fermentum, L. plantarum, L. brevis, L. casei, L. vaginalis, L. delbrueckii, L. salivarius, L. reuteri,* and *L. rhamnosus* [2, 5, 9–16]. As more studies are performed on the vaginal organisms in healthy women, it is possible that some women will be identified, who do not have a lactobacilli-dominated microbiota [17]. However, until we know more about the dynamics of such a population, and are sure that it does not increase the risk of the disease, lactobacilli will remain the organisms of most importance to vaginal health.

Factors such as hormonal changes (particularly estrogen), vaginal pH, and glycogen content can all affect the ability of lactobacilli to adhere to epithelial cells and colonize the vagina [16]. The menstrual cycle can also cause changes in the vaginal microbiota, with high concentrations of estrogen increasing adherence of lactobacilli to vaginal epithelial cells [18]. With the decrease in estrogen levels associated with menopause, there is also a decrease in lactobacilli present in the vaginal tract of postmenopausal women [5, 11, 12, 19]. Postmenopausal women are also more susceptible to urogenital infections, supporting the theory that colonization of the vagina by commensal lactobacilli serves as a protection from these pathogens [19, 20]. Although the methods by which these organisms do this are still unclear, it appears to involve an ability to adhere to and to populate the vaginal epithelium and mucin layer, to inhibit pathogens from taking over [21–24], to reduce pathogen virulence [25, 26], and to modulate host defenses [27].

Hormone replacement therapy (HRT) alters the bacterial profile of the vaginal tract of postmenopausal women, and restores a lactobacilli-dominated state, as well as reduces the incidence of urinary tract infections (UTI) [19]. In a study of women taking combination conjugated equine estrogen and progesterone HRT, only 1 to 3 species of bacteria, mainly *Lactobacillus*, were detected in the vaginal mucosa of 87% of the women [5]. In postmenopausal women not receiving HRT, almost all subjects had vaginal mucosa populated with more than 1 organism, many of which had pathogenic potential such as *Bacteroides, Prevotella,* and *Gardnerella*, associated with bacterial vaginosis (BV), and *E. coli* and *Enterococcus,* associated with UTI [5].

While a vaginal tract dominated by lactobacilli appears to protect the host against some vaginal infections, it does not fully prevent colonization by other species. Pathogens are still able to coexist with these commensal organisms, as shown by Burton and Reid [10], where *G. vaginalis*, a pathogen associated with BV, was detected in a vaginal sample which also contained a species of *Lactobacillus*. Interestingly, *G. vaginalis* was displaced beyond detectable limits for 21 days, following a single intravaginal instillation of probiotic lactobacilli [11]. As more and more studies are uncovering the diversity microbiota of the vagina, it seems apparent that the balance between a healthy and diseased state involves some sort of equilibrium or see-saw effect, which can swing in either direction depending on a number of factors, such as hormone levels, douching, sexual practices, as well bacterial interactions and host defenses [20, 21].

Witkin et al. [28] have proposed that innate immunity plays an important role in the switch to BV from a healthy state. The mechanism they propose is through microbial-induced inhibition of Toll-like receptor expression and/or activity blocking proinflammatory immunity, as well as a lack of 70-kDa heat-shock protein production, and a deficit in vaginal mannose-binding lectin concentrations decreasing the capacity for microbial killing. Three recent studies have provided further insight into the host's role. In a study of women susceptible to UTI, it was discovered that immunological defects in peripheral blood coexisted with a persistently aberrant microbiota (Kirjavainen et al. [29]). In postmenopausal women, BV was associated with apparent reduced expression of host antimicrobial factors [30]. When probiotic *L. rhamnosus* GR-1 was administered to the vagina of premenopausal women, it resulted in 3 536 gene expression changes and increased expression levels of some antimicrobial defenses [31].

## 2. NONSEXUALLY TRANSMITTED INFECTIONS OF THE VAGINAL TRACT AND INTERFERENCE BY LACTOBACILLI

Pathogenic organisms are able to infect the vagina, with BV, yeast vaginitis, and UTIs causing an estimated one billion or more cases per year [32–35]. While there is some evidence that the causative organisms can be transmitted by sexual partners, these conditions will be discussed here as nonsexually transmitted. Other reviews adequately cover sexually transmitted infections [36, 37].

Yeast vaginitis is characterized by white discharge, local itching, and irritation. The majority of cases are caused by *Candida albicans*, but *C. glabrata*, *C. krusei*, and *C. tropicalis* can be problematic [35]. It is diagnosed by microscopic detection of dense numbers of yeast cells on a vaginal smear, and by physical examination and the presence of a white, mucous-like yeast discharge. Of note, lactobacilli are often found in patients with yeast vaginitis, therefore, the induction of infection does not appear to require the yeast displacing or killing off the lactobacilli.

Urinary tract infections occur when pathogenic bacteria ascend from the vagina and replicate on, and sometimes within, the bladder urothelium [32, 38, 39]. These infections are frequent among women, with an estimated 50% suffering at some time in their life. Symptoms and signs include suprapubic pain, dysuria, pyuria, frequency and painful micturition, and occasionally hematuria. Asymptomatic bacteriuria is also a common occurrence, particularly amongst the elderly. The most frequent pathogen is *E. coli*, followed by *Enterococcus faecalis,* and *Staphylococcus saprophyticus* [39]. Diagnosis can be achieved by presence of symptoms and signs, and urine samples containing over 10^3^ organisms/mL of the pathogens. In a portion of patients, the *E. coli* invade the bladder epithelium and form dense biofilms that are recalcitrant to antibiotics [40]. In women with no history of UTI, their vagina and perineum is most commonly colonized by lactobacilli [20], while in women with recurrent UTI there is an inverse association between lactobacilli and *E. coli* [41], suggesting that lactobacilli play a role in preventing infection.

The most common urogenital disorder in women of reproductive age is BV, a condition discussed above. The vaginal microbiota of BV patients typically contains a broader range of species than found under healthy conditions, with *Atopobium vaginae*, *Bacteroides* spp., *Gardnerella vaginalis*, *Mobiluncus*, *Megasphera*, *Mycoplasma hominis*, *Peptostreptococcus*, and *Prevotella* being particularly prevalent [3, 42–46]. BV is associated with multiple species of bacteria that occur in 90% of the cases, and essentially consists of an elevated vaginal pH (>4.5) and depletion of lactobacilli. It affects women of all age groups, and is often asymptomatic [47]. When symptoms and signs do occur, they include fishy odor, discharge, and vaginal pH above 4.5 [48]. Indeed, this formed the basis of the often-used Amsel criteria for BV diagnosis: presence of at least 3 of the following criteria: (1) release of an amine or fishy odor upon addition of 10% potassium hydroxide, (2) a vaginal pH higher than 4.5, (3) detection of at least 20% of clue cells (which are vaginal cells colonized by Gram-negative rods), and (4) a milky homogeneous vaginal discharge [48]. A Gram-staining method called the Nugent score has also been used [8]. It comprises a scoring system based on the morphology of bacteria present in vaginal swab samples. A normal score is given to samples showing predominantly Gram-positive rods indicative of lactobacilli, while the presence of predominantly small and curved shaped Gram-negative rods and Gram-positive cocci, along with the absence of lactobacilli, is indicative of BV. The BVBlue test is another kit used to diagnose BV, and works by detecting sialidase produced by pathogens associated with the condition [49, 50]. Of note, aerobic vaginitis has also been described in which the vagina is colonized by organisms such as *E. coli* and enterococci [51]. During pregnancy, BV can increase the risk of preterm labor and low birth weight [52, 53]. Other problems associated with BV include pelvic inflammatory disease, UTI, and increased susceptibility to sexually transmitted diseases, including HIV [54–57].

The organisms associated with BV form dense biofilms on the vaginal epithelium, and these are associated with increased resistance to lactobacilli-produced lactic acid and hydrogen peroxide (H_2_O_2_) which are normally antagonistic to planktonic organisms [58]. The biofilms are also able to induce host expression of certain inflammatory factors, such as IL-1 and IL-8 [59]. It is not currently known whether the production of H_2_O_2_ by lactobacilli has a clinically protective role against BV. The increased prevalence of H_2_O_2_-peroxide producing vaginal lactobacilli in healthy women has been given as a reason to believe that it is a protective factor [60], however, those studies used culture to recover the lactobacilli, and arguably had they used nonculture methods, *L. iners* would have been the most commonly isolated and it does not appear to produce H_2_O_2_. It is possible to isolate *L. iners* by culture, but it requires selective media and extensive incubation. The same group found that women with the H_2_O_2_-producing vaginal *L. crispatus* or *L. jensenii* had a significantly lower incidence of BV than women with a different vaginal flora [14]. However, Alvarez-Olmos et al. [61] and Rosenstein et al. [62] found H_2_O_2_-producing lactobacilli in 85% and 91.7%, respectively, of women with BV. It could be argued that the high prevalence of H_2_O_2_-producing lactobacilli shows that this compound is not protective [32]. Either way, it is difficult to make a definitive conclusion.

McLean and McGroarty [63] conducted an in vitro study showing that increasing culture pH reduced the bacteriostatic effects of *L. acidophilus* on *G. vaginalis* NCTC 11292 by 60%; a 30% reduction in bacteriostatic effects was seen when catalase was introduced to degrade H_2_O_2_. Klebanoff et al. [64] found that the toxicity of H_2_O_2_-producing lactobacilli was inhibited by the presence of catalase but lactobacilli that do not produce H_2_O_2_ were not affected. High concentration of H_2_O_2_-producing lactobacilli inhibits the growth of both *G. vaginalis* and *Bacteroides bivius*. However, low concentrations of H_2_O_2_-producing lactobacilli must be combined with myeloperoxidase and chloride in vaginal mucus, to be toxic toward *G. vaginalis*, with a maximum toxicity in a pH range of 5 to 6. A pH of ≤4.5 inhibited the growth of *G. vaginalis* on its own and this effect increased with the addition of the above combination. Suffice to say, H_2_O_2_ is likely one of several factors involved in competition with other organisms in the vagina.

## 3. PROBIOTICS TO PREVENT AND TREAT UROGENITAL INFECTIONS

As antimicrobial treatment of urogenital infections is not always effective, and problems remain due to bacterial and yeast resistance, recurrent infections [65, 66], as well as side effects, it is no surprise that alternative remedies are of interest to patients and their caregivers. It is assumed that recurrences are due to antimicrobials failing to eradicate the pathogens, perhaps because of biofilm resistance, or that the virulent organisms come back from their source (the person's gut, or a sex partner) and attack a host whose defenses are suboptimal. Young girls who suffer from UTI are more likely to have repeated episodes in adulthood, and overall many UTI, BV, and yeast vaginitis patients will have a recurrence [21, 67]. Recurrent infection may also be due to the elimination of the commensal organisms in the vagina by the antimicrobial, thereby increasing susceptibility to recolonization by pathogens [68, 69]. This is one of the main reasons for considering the use of probiotics, to replenish the commensal microbes as a way to lower the risk of reinfection. In a study of 120 children with persistent primary vesicoureteral reflux, *L. acidophilus* treatment daily was as effective as trimethoprim/sulfamethoxazole in reducing the rate of UTI (*P* = .926), suggesting that probiotics could provide a prophylactic option [70].

The route of delivery of probiotic lactobacilli has intuitively been via direct instillation into the vagina. For example, the weekly application of *L. rhamnosus* GR-1 and *L. fermentum* B-54 was shown to reduce UTI recurrences from an average of 6 to 1.6 per year [71]. The ability of a given strain of lactobacilli to adhere to vaginal cells was considered an advantage in temporarily populating the vaginal [71, 72] and creating an environment conducive to the restoration of the host's indigenous lactobacilli rather than a return of pathogens. The adhesion of lactobacilli to the uroepithelium varies among species and strains, as shown by in vitro studies [72], and may be mediated by glycoprotein and carbohydrate adhesins binding to glycolipid receptors [73]. Still, it is unclear the extent to which a difference in in vitro adhesion, say of 10 per cell, means that an organism will succeed or fail to protect the host if instilled into the vagina. Thus, adhesion per se is not the definitive criteria to predict success. Once administered in a viable count of one billion or more, *L. rhamnosus* GR-1 and *L. reuteri* (formerly *fermentum)* RC-14 have been found to be detectable for three weeks or more, depending on the host [74, 75]. This implies a correlation between in vitro adherence and in vivo presence.

The concept of delivering lactobacilli orally to repopulate the vagina was first reported in 2001 [76], and based upon the question “if urogenital pathogens can do this, why cannot lactobacilli”? The organisms were delivered in a milk base and shown to be recovered from the rectum [77]; therefore supporting the concept that ingested strains could pass through the intestine, reach the rectum, and potentially ascend to the vagina. This was confirmed independently by others [78].

In order to conduct clinical studies with the view of providing more women with access to these strains, a two-year shelf life capsule formulation was then developed and used successfully in a number of studies. An oral dose of over one billion organisms per day was found to maintain a lactobacilli-dominated vaginal presence [79]. The time for this intervention to affect the vaginal tract is obviously longer than direct vaginal instillation, and will depend on viability of the strains as they pass through the stomach and gut [78]. In addition, the load of lactobacilli that can be delivered this way is clearly lower than via vaginal administration. However, an advantage of the oral approach may be the ability of the lactobacilli to reduce the transfer of yeast and pathogenic bacteria from the rectum to the vagina [80], which could potentially lower the risk of infection. In that randomized, placebo-controlled trial of 64 healthy women, 37% of the patients in the *L. rhamnosus* GR-1 and *L. reuteri* RC-14 probiotic group had a lactobacilli-dominated normal vaginal microbiota restored from a BV vaginal flora compared to 13% in the placebo group (*P* = .02). At both the 28-day and 60-day test points, women in the lactobacilli treatment group had a greater number of vaginal lactobacilli than women in the control group (*P* = .08 and *P* = .05, resp.) as shown by microscopy and culture. The ability of this oral probiotic therapy to create a lactobacilli normal flora and convert some subjects from a BV status to normal [79] goes beyond the proof-of-concept stage and provides a method for women to help maintain vaginal health. Failure of *L. rhamnosus* GG to be effective, at least in one small study [79], emphasizes the strain-specific aspects of probiotic use. Thus, one cannot and should not utilize the data from one strain to infer that another untested strain will provide the same benefits.

The mechanisms whereby lactobacilli function as anti-infective defenses are still not fully understood. As discussed above, this may involve production of antimicrobial factors [81], and maintenance of a vaginal pH of ≤4.5. It could also be due to biosurfactants which alter the surrounding surface tension and reduce the ability of a wide range of pathogens to adhere [82, 83]. This might explain the relatively sparse coverage of epithelial cells noted in healthy women [8]. In addition, lactobacilli have been shown to bind (coaggregate) some pathogens and this may be a means to block their adhesion, kill them through production of antimicrobials, and prevent their spread to other areas of the vagina and bladder [84]. Among 10 strains of lactobacilli being evaluated for use in a probiotics tablet, Mastromarino et al. [85] found, in vitro, that *Lactobacillus gasseri* 335 and *Lactobacillus salivarius* FV2 were able to coaggregate with *G. vaginalis*. When these strains of lactobacilli were combined with *Lactobacillus brevis* CD2 in a vaginal tablet, adhesion of *G. vaginalis* was reduced by 57.7%, and 60.8% of adherent cells were displaced. Boris et al. found that the adherent properties *G. vaginalis* were similarly affected by *Lactobacillus acidophilus* [73].

It has been known for some time that *Lactobacilllus* produce bacteriocins that can inhibit the growth of pathogens, including some associated with BV, such as *G. vaginalis* [86]. Only relatively recently has a study shown in animals that bacteriocin production might have an effect in vivo. A stable mutant of *Lactobacillus salivarius* UCC118 that did not produce a specific bacteriocin was unable to protect mice against *Listeria* intestinal infection, while the wild type did, thereby leading the authors to conclude that bacteriocin production can be a primary mediator of anti-infective defense [87].

Relatively few studies have attempted to prevent urogenital infection using probiotics. Shalev et al. [88] assessed 46 premenopausal women with ≥4 episodes of BV and/or vaginal candidiasis in the previous year, to compare the recurrence of BV using a probiotic yoghurt versus one that was pasteurized. Patients were not receiving long-term antibiotics or immunosuppressive therapy and had not consumed yoghurt prior to the commencement of the study. They were randomly assigned to one of two treatment groups and ingested 150 mL of either pasteurized yoghurt (*n* = 23) or yoghurt containing *L. acidophilus* at > 1.0 × 10^8^ colony-forming units (*n* = 23). Yoghurt was consumed daily for two months followed by two months of no yoghurt. There was a 60% reduction in BV episodes among patients consuming probiotic yoghurt after one month while only a 25% reduction occurred in subjects who received pasteurized yoghurt (*P* = .004). After two months of yoghurt consumption, the results were similar; however, 25% of patients from both groups had left the study. Product integrity was only assessed prior to the study and no adverse effects were reported.

Neri et al. [89] studied 84 women in the first trimester of pregnancy to observe the effects of probiotic-containing yoghurt on BV. The subjects were randomized to one of three treatment groups: inserting a tampon containing 5% acetic acid (*n* = 32), a 10 to 15 mL vaginal douche containing > 1.0 × 10^8^ colony-forming units/mL of *L. acidophilus* (*n* = 32), or no treatment (*n* = 20). Both active treatments were administered twice a day for one week. Amsel criteria (three of five findings: release of an amine fishy odor; release of amine odor after the addition of 10% potassium hydroxide; vaginal pH greater than 4.5; clue cells in the vaginal fluid; milky homogenous vaginal discharge) were absent in 88%, 38%, and 15% of subjects who received intravaginal lactobacilli, acetic acid tampons, and placebo, respectively, after 30 days. There was a significant difference in the cure rate between probiotic and control groups (*P* < .005), and lactobacilli and acetic acid groups (*P* = .004).

Fredricsson et al. [90] conducted an open-label trial to compare the cure rates of 61 women with BV given one of four intravaginal products. Patients were diagnosed with BV if ≥3 Amsel criteria were present. Each of the four treatments that patients were randomized to receive was administered twice a day for seven days: 5 mL of fermented milk containing between 5.0 × 10^8^ and 2.0 × 10^9^ colony-forming units/mL of *L. acidophilus* NCDO 1748 (*n* = 13), 5 mL of acetic jelly (*n* = 15), 5 mL of estrogen cream (*n* = 16), or 500 mg metronidazole vaginal tablets (*n* = 15). BV was considered to have been cured if ≤1 Amsel criterion was present at 4 and 8 weeks. After both 4 and 8 weeks from the initiation of treatment, the cure rates in the metronidazole, acetic acid, probiotic, and estrogen groups were 93%, 18%, 7%, and 6%, respectively; no statistical analysis was reported. In this case, the so-called probiotic was not effective. No information about the strain was provided.

The cure rates of BV in 57 women with a mean age of 24 were studied following treatment with either “probiotics” or placebo in a double-blind trial [91]. Subjects were randomized to receive either a vaginal suppository containing 1.0 × 10^8-9^ colony-forming units of *L. acidophilus* (*n* = 28) or placebo (*n* = 29). The vaginal suppositories were administered twice a day for 6 days. Symptom resolution, which was not clearly defined, was used to evaluate the cure of BV. At 7–10 days after the commencement of treatment, BV symptoms were absent in 57% of women in the probiotic group and 0% of women in the placebo group (*P* < .005). After 20 to 40 from the initiation of treatment, the cure rate in the probiotic group fell to 21% and remained at 0% in the placebo group (p = NS). This poorly conceived study is hard to interpret and is insufficient to verify efficacy of the product.

Eriksson et al. [92] studied how lactobacilli augmented antibiotics in curing BV through a double-blind, placebo-controlled trial including 187 women with a median age of 32 over two menstrual periods. Open-label treatment with 100 mg/d of clindamycin was administered to all patients for 3 days. The subjects were then randomized to one of two treatment groups which required at least five tampons to be inserted during the next menstrual period. The treatment groups were placebo tampons (*n* = 96) and tampons impregnated with *L. fermentum, L. gasseri*, and *L. rhamnosus* at 1.0 × 10^8^ colony-forming units per tampon (*n* = 91). Cure rates of BV were assessed by the absence of Amsel criteria after the second menstrual period in both the probiotic and placebo groups, and found to be 56% and 62%, respectively, (p = NS). Infection with *Candida* was reported in 14.3% of subjects in the probiotic group and 13.5% of patients in the placebo group. The viable number of bacteria per tampon diminished to 10^6^ colony-forming units by the end of the study. In short, this product was not successful. The rationale for administering lactobacilli during menses could be questioned, as it exposes the users' blood stream directly to the organisms, and the flushing effect of menstruation may be nonconducive to lactobacilli repopulating the vagina.

A comparison of intravaginal probiotics and metronidazole gel in treating 40 women (ages 18 to 50) with BV was conducted by a single-blind study by Anukam et al. [93]. The presence of ≥3 Amsel criteria, a Nugent score of ≥7, and a positive sialidase test led to a diagnosis of BV. Patients were randomized to one of two treatment groups for five days. They either inserted an intravaginal capsule with *L. rhamnosus* GR-1 and *L. reuteri* RC-14 at 1.0 × 10^9^ colony-forming units nightly (*n* = 20) or applied a 0.75% metronidazole gel twice daily (*n* = 20). A Nugent score of ≤3 at 30 days indicated a cure of BV. A BV cure rate of 88% in the probiotic group and 50% in the metronidazole group was found (p = NS). Treatment was prematurely discontinued by patients in both the metronidazole and probiotic groups at 10% and 15%, respectively. This study, albeit small in size, showed the potential of probiotics to cure BV.

The efficacy of combining probiotics or placebo with oral metronidazole was assessed in 125 women aged 18 to 44 [94]. Oral metronidazole was administered at 500 mg twice daily to all patients for 7 days, and they were randomized to receive twice-daily oral capsules containing either a placebo (*n* = 60) or *L. rhamnosus* GR-1 and *L. reuteri* RC-14 at 1.0 × 10^9^ colony-forming units (*n* = 65) for a total treatment duration of 30 days. At the end of 30 days, BV was considered absent if the patient had a negative sialidase test and a Nugent score of <3. This was the case in 40% of placebo and 88% of probiotic subjects (*P* < .001). If an intermediate Nugent score was regarded as “cure of BV”, the cure rate was 100% with metronidazole and probiotics versus 70% with metronidazole and placebo. This study is important as it implies that probiotics can augment the effects of antibiotics in treatment of disease. Further studies have confirmed this effect, but are awaiting publication.

## 4. POSSIBLE NEGATIVE EFFECTS OF PROBIOTIC USE

Annually, over one billion doses of probiotics are administered worldwide, and those administered for urogenital health have been well tolerated [11, 75, 93–96]. In addition, the mouth, gastrointestinal tract, and female genitourinary tract are inhabited by *Lactobacillus* [96]. Yet, endocarditis and bacteremia caused by lactobacilli are extremely rare. Most cases occur in patients with chronic diseases or debilitating conditions that provide direct access to the bloodstream from a leaky gut. Only 1.7% of 241 cases of bacteremia, endocarditis, and localized infections associated with *Lactobacillus* that were investigated by Cannon et al. were considered to have a possible link with heavy consumption of dairy products [97]. Only one case had a *Lactobacillus* isolate that was indistinguishable from a probiotic strain. There was no connection between the species of *Lactobacillus* isolated and the type of infection or mortality. A recent study that directly instilled a six-strain bacterial product into the intestine of patients with severe, potentially fatal pancreatitis portrayed probiotics as being dangerous [97]. However, the product had never been proven to be probiotic, it was administered as a drug unlike 99.9% of probiotics, the randomization process led to patients with multiorgan failure being given large doses of live bacteria, and the authors failed to provide a rationale for the study in an appropriate animal model. All this led to unwarranted adverse publicity for the field of probiotics [98].

Nevertheless, safety of probiotic use must continually be monitored and considered when doing clinical studies. The potential for transfer of antibiotic resistance is one factor to consider, although it remains to be proven that probiotics have contributed in any way to drug resistance, or disease. Rather, the overuse of antibiotics, especially in livestock feed and long-term prevention of infection, remains a root cause of the increasing concerns over drug resistance. Efforts to substitute prophylactic antibiotics with probiotics, especially in children with recurrent UTI [70] and perhaps some patients preparing to undergo surgery [99], are worthy of pursuit.

## 5. CONCLUSION

Molecular methodologies are providing a greater understanding of the dynamic microbial presence, both short and long term, in the vagina. The defenses of the host which include some of these microbes perform a remarkable function given the opportunity of pathogens to cause infection. The use of probiotic lactobacilli to prevent infection has a good rationale, and an excellent safety record, but so far only a few strains have been clinically proven to be effective, in particular to prevent BV. It is critically important that strains be characterized and tested clinically using the delivery system of choice (oral, vaginal, dried powder, or in suspension). An advantage for women is that they can self-administer the probiotics. Many more studies are needed to optimize the defensive properties of the vaginal microbiota, but the potential remains that the health of many women can be improved by probiotic intervention.
